# Effects of Different Patient and Prosthetic Variables on Marginal Bone Behavior in Dental Implants: A Clinical Retrospective Study

**DOI:** 10.3390/medicina61061041

**Published:** 2025-06-05

**Authors:** Sergio Alexandre Gehrke, Antonio Scarano, Felice Lorusso, Thalles Yurgen Balduino, Marco Aurélio Bianchini

**Affiliations:** 1 Department of Implantology, Bioface/Postgrados en Odontología/Universidad Catolica de Murcia, Montevideo 11100, Uruguay; 2 Department of Biotechnology, Universidad Católica de Murcia, 30107 Murcia, Spain; 3 Department of Innovative Technologies in Medicine & Dentistry, University of Chieti-Pescara, 66013 Chieti, Italy; ascarano@unich.it (A.S.); drlorussofelice@gmail.com (F.L.); 4 Department of Dentistry, Center for Education and Research on Dental Implants (CEPID), Federal University of Santa Catarina (UFSC), Florianópolis 88040-900, SC, Brazil; thallesbalduino@hotmail.com (T.Y.B.); marco.bianchini@ufsc.br (M.A.B.)

**Keywords:** clinical prospective study, dental implants, Morse taper connection, marginal bone level, transmucosal abutment, emergence profile, peri-implant bone stability

## Abstract

*Background and Objectives:* This study aimed to evaluate marginal bone level (MBL) changes in implant-supported rehabilitation based on patient demographics, implant location, transmucosal abutment height, and crown emergence profile. *Materials and Methods*: A total of 50 patients (28 females and 22 males), with 111 implant–abutment (IA) sets, were analyzed. The mean age was 65.2 ± 10.9 years (range: 33–81). Implants were placed in the maxilla (68.5%) and mandible (31.5%), with an average evaluation period of 12.7 ± 4.1 months. MBL changes at mesial (MBLm) and distal (MBLd) sites were recorded and analyzed based on sex, age, implant location, abutment transmucosal height, and crown emergence profile. Statistical comparisons were performed using Bonferroni’s multiple comparison test and one-way ANOVA with Tukey’s post hoc test. *Results*: MBL changes did not show significant differences based on sex (*p* > 0.05) or age group (*p* > 0.05). However, maxillary implants exhibited greater bone loss than mandibular implants, though this difference was not statistically significant. Transmucosal abutment height (TMh) significantly influenced MBL, with taller abutments (TMh3: −1.07 ± 0.93 mm) showing less bone loss than shorter abutments (TMh1: −2.11 ± 1.82 mm) (*p* < 0.05). Crown emergence profile also affected MBL, particularly in the distal emergence profile, where design 1 exhibited the least bone loss compared to designs 2 and 3 (*p* = 0.0176). *Conclusions*: The study findings suggest that transmucosal abutment height and crown emergence profile significantly influence peri-implant bone stability. Transmucosal abutment height (> 2.5 mm) was associated with reduced bone loss. Further research is recommended to evaluate the long-term effects on peri-implant bone maintenance.

## 1. Introduction

Dental implants have become a widely accepted solution for replacing missing teeth, providing long-term functionality and aesthetic benefits. Over time, the procedure has become more predictable, with high success rates [1,2]. However, the success of implant-supported rehabilitation depends on various general and local factors that must be considered during the planning phase. Marginal bone loss (MBL) is a dynamic process that may begin prior to prosthetic loading, and controlling it is one of the primary concerns. Excessive bone resorption can compromise implant longevity, leading to functional and aesthetic complications [3].

Several factors have been associated with MBL, including patient-related variables such as age and sex, implant site (maxilla vs. mandible), and prosthetic components, such as the transmucosal abutment height and crown emergence profile. The transmucosal abutment, which connects the implant to the prosthetic restoration, has been suggested to influence peri-implant bone preservation [4,5]. Similarly, the design of the crown emergence profile can affect biomechanical forces around the implant, potentially impacting bone stability [6,7].

In addition to the factors mentioned above, the type of prosthetic connection—screw-retained versus cement-retained crowns—has also been considered as a potential influence on peri-implant tissue behavior. Although this topic has generated considerable discussion, recent evidence suggests that the choice between these two retention types may not significantly affect marginal bone loss (MBL). Recent studies [8,9] concluded that marginal bone changes do not differ significantly between cement-retained and screw-retained implant-supported crowns.

The shape and design of the crown, especially its cervical geometry, play a crucial role in the long-term success of implants, particularly in relation to marginal bone stability [7,10]. However, designing a crown for an implant presents challenges. For instance, the implant/abutment (IA) interface is typically circular, while most dental crowns have different shapes. Additionally, the diameter of the IA is often much smaller than that of the natural crown being rehabilitated.

The cervical design of the crown refers to the interface between the crown and implant abutment, which is crucial for promoting an adequate biological response in the peri-implant tissues. Factors such as the emergence profile, the cervical diameter of the crown, and the transition between the prosthetic structure and soft tissues significantly influence tissue adaptation, the distribution of masticatory forces, and, consequently, the maintenance of marginal bone levels [11]. Choosing the right cervical prosthetic design can prevent complications like marginal bone loss and peri-implant inflammation, which directly affect implant longevity [12].

Studies show that an adequate cervical prosthetic design can reduce the risk of mechanical overload, one of the main causes of bone loss around implants [13]. Additionally, the shape of the crown and the choice of material used in cementation or screwing are critical to therapeutic success [14]. A prosthetic connection that ensures uniform force distribution plays a vital role in preserving marginal bone, contributing to long-term success [15]. Therefore, understanding how the cervical design of the single crown impacts peri-implant health and the maintenance of marginal bone levels is essential for clinical practice. Proper manipulation of these parameters results in better aesthetic and functional outcomes, ensuring greater durability and implant success.

The shape of prosthetic components (implant, abutment, crown) is a key determinant of long-term treatment success. Designs that favor adequate soft tissue adaptation and ensure balanced occlusal force distribution promote the preservation of the marginal bone. Retrospective studies have been valuable in analyzing the variables involved and understanding the impact of cervical design on bone health around implants. By examining clinical outcomes from past treatments, retrospective analysis offers valuable insights into the long-term effects of different single crown configurations on marginal bone stability.

Despite extensive research, there remains a need for a deeper understanding of how these variables interact and influence peri-implant bone health. This study aims to evaluate MBL changes in implant–abutment (IA) sets based on patient demographics, implant location, abutment height, and crown emergence profile. Through this analysis, the study seeks to provide valuable insights into optimizing prosthetic design for improved implant success and long-term peri-implant bone preservation, ultimately contributing to clinical practice in implantology. The null hypothesis was that there would be no statistically significant differences in marginal bone level changes according to patient demographics, implant location, transmucosal abutment height, or crown emergence profile.

## 2. Materials and Methods

### 2.1. Patients and Implants

All patients selected and included in this study were restored with implant fixed rehabilitation. In all cases, the same implant systems were used (Maestro implant, Implacil/Osstem, São Paulo, Brazil) between September 2019 and September 2022. The present study obtained approval from the Research Ethics Committee of the Federal University of Santa Catarina (UFSC), under Opinion Number: 5,972,205 and CAAE: 67753123.6.0000.0121, in accordance with Resolution No. 466, dated 12 December 2012. The Declaration of Helsinki (1975, updated 2013) was followed. Furthermore, according to clinic standards, all patients agreed to and signed an informed consent authorizing data collection.

The sample for this study consisted of 50 patient medical records retrieved from the archives of the Smiling Dental Clinic (Florianópolis, Brazil). The cohort included both male and female patients who collectively received 111 implants. These implants supported various prosthetic rehabilitations, including both single and multiple prostheses, which were fixed through either cement or screw retention.

Table 1 shows the inclusion and exclusion criteria.

All evaluated implants presented an internal Morse taper connection, 3.5 mm in diameter, with lengths of 7, 9, 11, and 13 mm. The implants were placed in both arches (maxilla and mandible) and in anterior and posterior areas. The opposing arches exhibited a variety of dental configurations, including natural teeth, fixed or removable partial dentures, implant-supported dentures, and complete dentures. All installation site conditions were included: immediate implant placement after tooth extraction or healed site, implant placement associated with bone regeneration or not and, immediate provisionalization or not.

### 2.2. Data Collected from Clinical Observation Files

Data related to patient age, sex, position of the rehabilitated tooth (anterior or posterior), arch (maxilla or mandible), crown installation period, and abutment transmucosal height were collected for analysis. In all the cases analyzed, the implants were reopened after 3 months of installation, as per the general rule for these cases. The definitive crowns were installed an average of 5 months after implant placement. Furthermore, regarding the transmucosal height (TMh) of the abutments evaluated, these were divided into 3 classes: TMh1, abutments with heights of 0.8 and 1.5 mm; TMh2, abutments of 2.5 mm; and TMh3, abutments ≥ 3.5 mm (3.5, 4.5 and 5.5 mm). Additionally, data were extracted from medical records concerning the duration of follow-up, as well as X-ray images acquired on the day of the implant-supported rehabilitation installation and subsequently at the last check-up at least one year after the installation of the prosthesis.

### 2.3. Data Collected from Radiological Images

**Figure 1:**
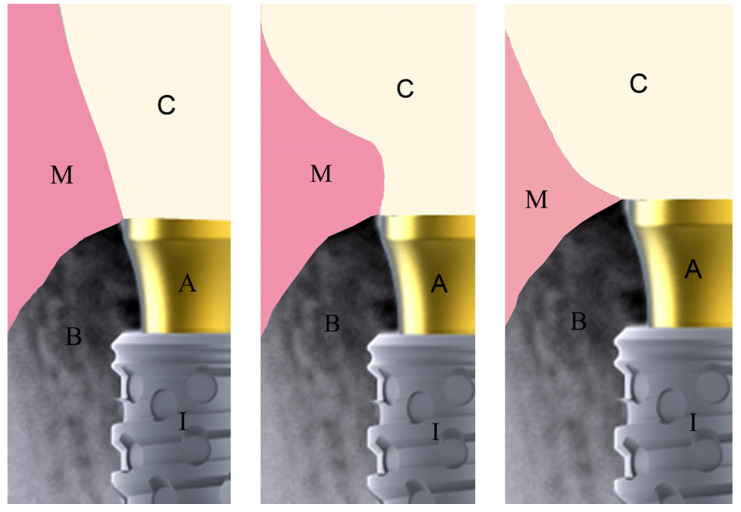
Schematic image of the 3 crown designs considered: designs 1, 2 and 3, respectively. M = mucosa, C = crown, A = abutment, I = implant, B = bone.

The emergence profile (EP) of the implant crowns was determined for each side of the crown (mesial and distal) on the radiographic images, and divided into 3 designs: design 1, where the crown design followed the diameter of the abutment in an almost straight line; design 2, where the crown design presented a concave shape after leaving the abutment base; and, design 3, where the crown design presented a convex shape after leaving the abutment base. Figure 1 schematically shows the different crown designs considered.

#### 2.3.1. Types of Radiological Images Used

All patients selected in the present study presented periapical radiography using the parallel cone technique with a Rinn alignment system (Insight Film Kodak, Carestream, Rochester, NY, USA) and a digital rigid film–object X-ray source coupled to a beam-aiming device (to achieve reproducible exposure geometry), in their medical records. The analyses were performed on radiographs taken immediately after the installation of the implant rehabilitation (C1), and on radiographs from the last clinical control with a minimum of one year of follow-up (C2).

#### 2.3.2. Processing of Radiological Images Using the Software

**Figure 2:**
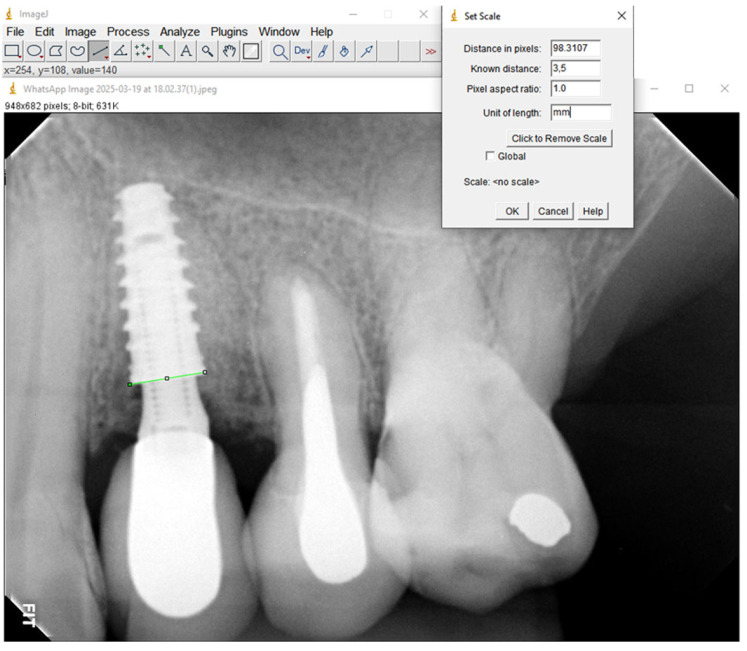
Image of the ImageJ software calibration using the implant diameter (green line) to realize the measurements.

The radiographic images obtained from each patient were meticulously analyzed utilizing ImageJ software version 1.49 (National Institutes of Health, Bethesda, MA, USA). Firstly, the known dimensions of the implant were utilized in combination with the set scale tool to convert the measurement units to millimeters and calibrate the software. This calibration of the image enabled accurate measurement assessments (Figure 2).

#### 2.3.3. MBL Distance Measurement

**Figure 3:**
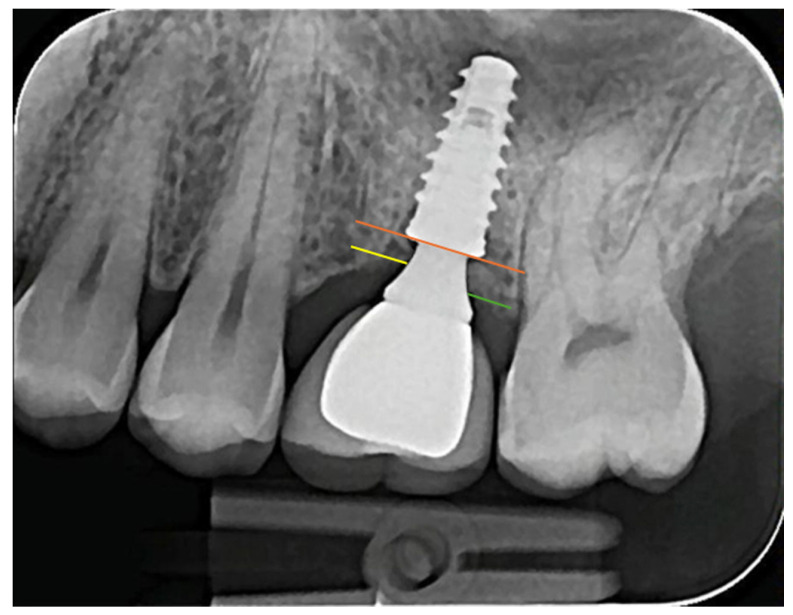
Representative image of the MBL measured in each radiography. The distance between the implant platform (orange line) to the first contact with the bone in the IA set in mesial (yellow line) represents the MBLm; in distal, the distance between the orange and green lines represents the MBLd.

Then, to analyze the parameter related to peri-implant marginal bone level (MBL), in the mesial (MBLm) and distal (MBLd) side of each implant, a line was drawn on the implant platform and another at the first contact of the bone tissue with the IA set in each side, as illustrated in Figure 3. These measurements were made on radiographs from time C1 (rehabilitation installation) and C2 (last control), and the resulting value was then calculated; that is, the difference obtained between the value of C1 and C2 (MBLr).

#### 2.3.4. Emergence Profile Measurement

**Figure 4:**
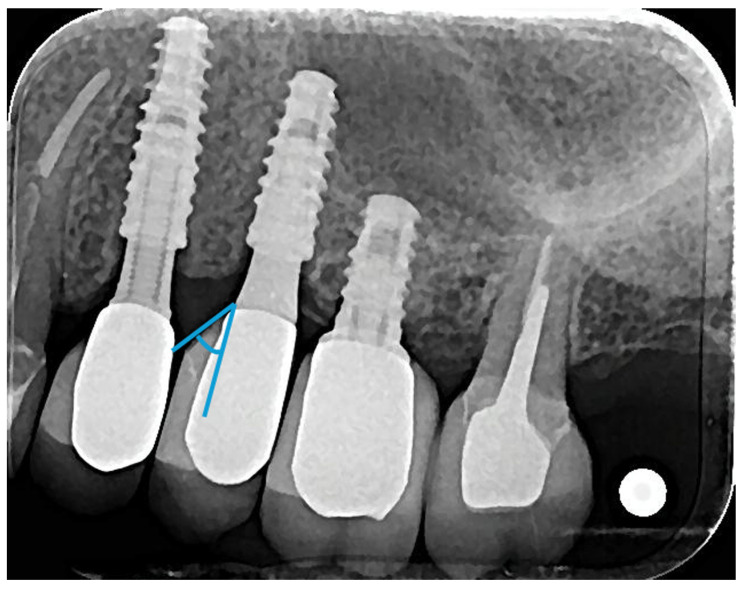
Representative image of the measurement of the emergence angle (EA) from the abutment platform. A blue line was drawn parallel to the long axis of the implant and the other with the crown design.

Regarding the emergence profile (EP) measurements, considering that all implants were installed in a subcrestal position of approximately 2 mm, as recommended by the manufacturer, the transition from this position to the desired level for the start of the crown was made by the abutment, and the angle was measured from the abutment platform (Figure 4). Then, a straight line was drawn from the abutment platform following the long axis of the implant, and another line tangential to the crown. The angle formed between these lines was subsequently measured. At each designated point (M and D), two measurements were obtained, and the average value was calculated.

### 2.4. Examination Data and Error Calculation

The same examiner (S.A.G.), who has extensive experience in dental implants and image analysis, performed all the measurements, which were repeated three times in each mesial and distal position. After two weeks, the same measurements were taken again to calculate the average, which served as the reference value and helped determine the estimated error margin. The intra-examiner error was calculated to be 0.05 mm on average, indicating that the intra-operator error was not statistically significant (*p* = 0.18, 95% CI).

### 2.5. Statistical Analysis

Statistical analyses were conducted to compare MBL changes across different variables, including sex, age, implant location, transmucosal abutment height, and crown emergence profile. Data normality was assessed using the Shapiro–Wilk test, and parametric tests were applied where appropriate. Independent *t*-tests were used for two-group comparisons, while one-way ANOVA followed by Tukey’s post hoc test was applied for comparisons involving more than two groups. Bonferroni’s multiple comparison test was used to confirm significant differences.

Following reviewer recommendations, 95% confidence intervals (CIs) were included to enhance the interpretation of key findings. Multivariable analysis was not performed due to sample size limitations and to avoid overfitting. All data were analyzed using SPSS Statistics for Windows, Version 26.0 (IBM Corp., Armonk, NY, USA), with statistical significance set at *p* < 0.05.

As this was a retrospective study, a priori sample size calculation was not feasible. However, a post hoc power analysis was performed to evaluate the adequacy of the sample. The primary outcome considered was the marginal bone level (MBL) change. The effect size observed between the shortest (TMh1) and tallest (TMh3) abutment height groups was 1.04 mm, with a pooled standard deviation of 1.45 mm. Based on this effect size, the calculated statistical power exceeded 80%, indicating that the sample size was sufficient to detect clinically meaningful differences.

## 3. Results

The null hypothesis, which posited that there would be no statistically significant differences in marginal bone level (MBL) changes according to patient demographics, implant location, transmucosal abutment height, or crown emergence profile, was partially rejected. Statistically significant differences in MBL were observed based on prosthetic parameters, specifically the transmucosal abutment height and crown emergence profile (*p* < 0.05). However, no significant differences were found related to patient demographics, such as sex and age, or implant location (*p* > 0.05).

**Figure 5:**
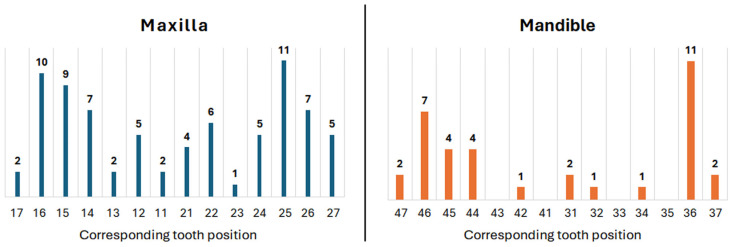
Bar graph showing the distribution (quantity) of IA sets by corresponding tooth position.

A total of fifty patients, encompassing 111 rehabilitations on implant/abutment sets, were included and analyzed. Of this total number of patients, 28 (56%) were female and 22 (44%) were male. However, of the total IA sets evaluated, 54 (48.7%) were in females and 57 (51.3%) in males. The mean and standard deviation age of the participants was 65.2 ± 10.9 years (ranging between 33 and 81 years). The mean period of evaluation was 12.7 ± 4.1 months. Seventy-six implants were installed in maxilla sites (68.5%), and 35 were installed in mandible sites (31.5%). The distribution (quantity) of IA sets by corresponding tooth position is graphically presented in Figure 5.

Table 2 shows the mesial and distal MBL measurements in the different variables and the statistical comparison between them.

Of the data presented in Table 2, only the variables related to the patient’s sex and age were statistically compared, as these presented a closer number of implants evaluated. In both cases, no significant differences were found for the MBLm and MBLd values (*p* > 0.05).

Regarding the analysis of the height of the transmucosal abutments evaluated, an average was taken between the MBLm and MBLd values, as no significant differences were found between these values (*p* > 0.05), resulting in the following values: −2.11 ± 1.82 mm for TMh1, −1.52 ± 1.27 mm for TMh2, −1.07 ± 0.93 mm for TMh3. However, when compared between the three proposed classes, they presented significant differences in the calculated MBLr values, as shown in Table 3.

**Figure 6:**
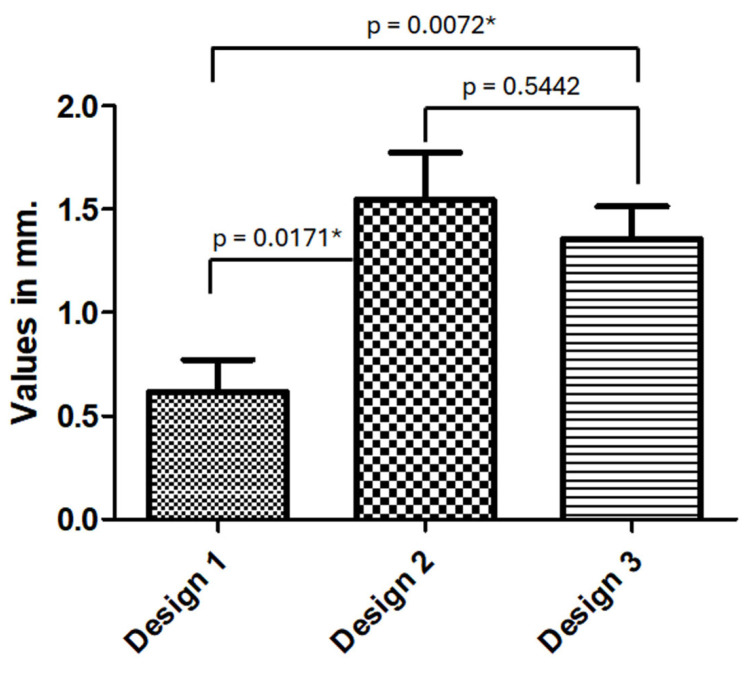
Bar graph showing the statistical differences between the MBLr values presented in the distal emergence profile. *: statistically significant difference (*p* < 0.05).

The values obtained regarding the cervical design of the crowns regarding the marginal bone level in distal and mesial positions and the values of the angle formed between the base of the abutment and the emergence of the crown are described and presented in Table 4. As there was only a statistical difference in the MBLr values between the designs in the distal position, the comparison between the designs is demonstrated in Figure 6.

## 4. Discussion

This retrospective clinical study evaluated the impact of various patient- and prosthetic-related variables on marginal bone level (MBL) changes in implant-supported rehabilitation. MBL changes in implant–abutment (IA) sets were based on various factors, including patient demographics, implant location, transmucosal abutment height, and crown emergence profile. The analysis of 111 IA sets from 50 patients provided insight into factors affecting peri-implant bone stability. The results suggest that while patient demographics such as sex and age did not show a statistically significant influence, prosthetic parameters—specifically, transmucosal abutment height and crown emergence profile—were significantly associated with peri-implant bone preservation. These findings contribute to a growing body of evidence underscoring the importance of prosthetic design in long-term implant success. The null hypothesis was partially rejected based on the statistical findings of the study. Although patient demographics (age and sex) and implant location (maxilla vs. mandible) did not show significant associations with changes in MBL, prosthetic factors, particularly the transmucosal abutment height and crown emergence profile, were found to significantly influence bone preservation. These findings suggest that implant design parameters play a crucial role in peri-implant bone health, supporting the idea that prosthetic factors, rather than patient demographics, are the primary determinants of MBL changes.

### 4.1. Influence of Demographic and Anatomic Variables

In agreement with previous studies [16,17,18], implants placed in the maxilla tended to exhibit greater MBL than those in the mandible, although the difference in our cohort did not reach statistical significance. This trend is often attributed to lower bone density in the maxilla, leading to reduced primary stability and increased bone remodeling during healing [16]. While our data did not demonstrate significant differences based on patient sex or age, other authors have reported mixed findings. For instance, some studies suggest males may experience higher MBL due to behavioral or anatomical differences [19], while others found younger patients exhibited increased bone remodeling due to higher metabolic activity [20]. In our study, the slightly higher MBL in younger patients was not statistically significant but may still be clinically relevant, warranting further investigation.

### 4.2. Prosthetic Factors: Abutment Height

One of the most relevant findings was the association between increased transmucosal abutment height and reduced MBL. Our results are in line with other studies [4,5], which demonstrated that abutments ≥2.5 mm in height create a more favorable soft tissue seal and reduce the impact of inflammatory infiltrate near the crestal bone. The significant differences in MBL observed between TMh1, TMh2, and TMh3 groups suggest that clinicians should prefer taller abutments when feasible. This approach may also be synergistic with the biological width concept, helping establish a more stable peri-implant mucosal barrier [21].

### 4.3. Crown Emergence Profile and MBL

The emergence profile of the prosthetic crown, particularly in the distal region, was another key factor associated with bone preservation. In our cohort, design 1 (straight profile) resulted in significantly less bone loss compared to designs 2 (concave) and 3 (convex). These findings are consistent with other studies [22,23], which found that excessive emergence angles (>30°) were associated with a higher risk of peri-implantitis and bone loss. Conversely, other studies reported more nuanced or contradictory results. For example, Cho et al. [24] found no significant influence of emergence angle on MBL when the angle remained under 30°, while Inoue et al. [25] suggested that moderate angles (20–40°) combined with conical abutment connections are optimal for peri-implant health.

The divergence in the literature likely reflects heterogeneity in study designs, follow-up durations, implant systems, and patient variables [26,27]. Nonetheless, our data contribute to the argument that careful design of the crown emergence profile—aimed at minimizing steep angles and excessive contour—can favorably impact bone stability.

### 4.4. Clinical Implications

The findings of this study suggest that factors such as implant location, transmucosal abutment height, and crown emergence profile play a crucial role in peri-implant bone maintenance. Clinicians should consider selecting abutments with the longest transmucosal portion possible to reduce marginal bone loss and optimize emergence profile designs to minimize peri-implant stress. While patient demographics such as age and sex did not show significant associations with MBL changes, further research with larger sample sizes may be needed to confirm these findings.

### 4.5. Limitations

While this study offers valuable insights, several limitations must be acknowledged:

1. Imaging Modality: MBL was assessed using periapical radiographs, which, while standardized using the parallel cone technique and digital calibration, may be less precise than cone-beam computed tomography (CBCT). Radiographs primarily capture 2D mesial–distal bone changes and may underestimate buccal or lingual bone loss [24].
2. Follow-Up Period: The average follow-up time of approximately 12.7 months, although clinically meaningful, does not allow conclusions to be drawn about long-term outcomes. Marginal bone remodeling is a dynamic process that may evolve beyond the first year after loading.
3. Sample Size and Study Design: Although 111 IA sets were evaluated, the number of implants in some subgroups (e.g., emergence profile designs) was limited, which could affect the statistical power. Moreover, as a retrospective study, inherent biases related to patient selection, data completeness, and lack of randomization may affect the generalizability of the results.
4. Confounding Variables: Despite efforts to control for systemic diseases and smoking, other factors such as oral hygiene, prosthetic material, occlusal loading, and surgical technique could also influence MBL and were not comprehensively analyzed.
5. Intra-Observer Reliability: While intra-examiner reliability was assessed (error margin: 0.05 mm), we did not perform inter-observer calibration and measurements.

### 4.6. Future Directions

Prospective, randomized studies with longer follow-up and 3D imaging techniques such as CBCT are necessary to validate our findings and clarify the complex interactions between prosthetic design, patient biology, and peri-implant bone response. In particular, studies comparing different abutment heights and emergence angles in a standardized clinical context could provide definitive guidance for clinical protocols.

## 5. Conclusions

This study evaluated the influence of patient and prosthetic factors on marginal bone loss in dental implants. The null hypothesis was partially rejected. No statistically significant differences were found for sex, age, or implant location. However, transmucosal abutment height and crown emergence profile significantly affected peri-implant bone stability. These findings emphasize the importance of prosthetic design in preserving marginal bone and ensuring long-term implant success.

## Figures and Tables

**Table 1:** Inclusion and exclusion criteria.

Inclusion Criteria	Exclusion Criteria
Individuals who had implant-supported rehabilitation installed at least one year before the initiation of the study	Individuals who had taken medications influencing bone remodeling within the preceding six months (e.g., bisphosphonates)
Individuals receiving either single or multiple implants	Pregnant or lactating women
Patients aged 18 years or older	Cases with missing or incomplete information
Implants featuring a 3.5 mm diameter and Morse taper connection	Physical disorders which would interfere with the ability to maintain oral health care
No systemic diseases (e.g., diabetes, osteoporosis, hypertension, etc.)	
Controlled periodontal conditions	
Individuals who smoked fewer than 10 cigarettes per day	

**Table 2:** MBLr values obtained for MBLm and MBLd (in mm) for different variables and their statistical analysis.

Independent Variables (in mm)	MBLm	MBLd	*p*-Value
General	(*n* = 111) −1.34 *±* 1.24	(*n* = 111) −1.29 *±* 1.22	0.3214
Female	(*n* = 54) −1.04 *±* 0.93	(*n* = 54) −1.16 *±* 0.91	0.7916
Male	(*n* = 57) −1.71 *±* 1.57	(*n* = 57) −1.41 *±* 1.44	0.1205
Age (33 to 64)	(*n* = 49) −1.48 *±* 1.42	(*n* = 49) −1.49 *±* 1.43	0.7411
Age (65 to 81)	(*n* = 62) −1.30 *±* 1.27	(*n* = 62) −1.13 *±* 0.99	0.3348
Maxilla	(*n* = 76) −1.67 *±* 1.44	(*n* = 76) −1.57 *±* 1.33	0.6879
Mandible	(*n* = 35) −0.72 *±* 0.72	(*n* = 35) −0.63 *±* 0.46	0.0885
Anterior	(*n* = 23) −1.84 ± 1.37	(*n* = 23) −1.79 *±* 1.29	0.8778
Posterior	(*n* = 88) −1.26 ± 1.31	(*n* = 88) −1.15 ± 1.17	0.2717

MBLm = mesial marginal bone level; MBLd = distal marginal bone level.

**Table 3:** Comparison between the abutments analyzed with different transmucosal heights using Bonferroni’s multiple comparison test.

Comparison	Mean Diff.	t	*p*-Value	95% CI of Diff.
TMh1 vs. TMh2	0.5949	2.166	0.0024	−0.06780 to 1.258
TMh1 vs. TMh3	1.039	4.182	0.0033	0.4396 to 1.638
TMh2 vs. TMh3	0.4441	2.322	0.0017	−0.01728 to 0.9056

**Table 4:** Analysis of the crown emergence profile in distal and mesial sides with MBLr and angle measurements. Statistical analysis using one-way ANOVA test, followed by Tukey’s post hoc test.

	*n* (%)	MBLr (±SD)	*p*-Value	F-Value	df	Angle (±SD)	*p*-Value	F-Value	df
D.E. profile			0.0176 *	3.913			<0.0001 *	10.70	
Design 1	19 (17.1%)	0.62 (±0.68)			2 (df 1)	14.74 (±5.65)			2 (df 1)
Design 2	33 (29.7%)	1.54 (±1.32)			108 (df 2)	23.26 (±13.25)			108 (df 2)
Design 3	59 (53.2%)	1.35 (±1.23)			110 (df 3)	31.05 (±15.98)			110 (df 3)
M.E. profile			0.0545	3.573			<0.0001 *	3.272	
Design 1	16 (14.4%)	1.54 (±1.72)			2 (df 1)	16.07 (±6.35)			2 (df 1)
Design 2	39 (35.1%)	1.78 (±1.51)			108 (df 2)	23.67 (±9.54)			108 (df 2)
Design 3	56 (50.5%)	1.06 (±0.98)			110 (df 3)	35.05 (±13.36)			110 (df 3)

D.E.: distal emergence; M.E.: mesial emergence; *: statistically significant difference; df: degrees of freedom; df 1: treatment (between columns); df 2: residual (within columns); df 3: total.

## Data Availability

The data that support the findings of this study are available from the corresponding author upon reasonable request.
